# Temporal Expression of Pelp1 during Proliferation and Osteogenic Differentiation of Rat Bone Marrow Mesenchymal Stem Cells

**DOI:** 10.1371/journal.pone.0075477

**Published:** 2013-10-16

**Authors:** Jing Wang, Shujun Song, Liang Shi, Qiang Zhu, Chuanchuan Ma, Xiaoqing Tan, Yin Ding, Zhongying Niu

**Affiliations:** 1 Department of Orthodontics, College of Stomatology, Forth Military Medical University, Xin Cheng District, Xi’an, China; 2 Department of Pathology and Experimental Medicine, 306 Hospital of PLA, Chao Yang District, Beijing, China; 3 Department of Stomatology, 306 Hospital of PLA, Chao Yang District, Beijing, China; 4 Department of Urological, General Hospital of People’s Liberation Army, Hai Dian District, Beijing, China; University of Nevada School of Medicine, United States of America

## Abstract

**Background:**

Osteogenic induction and bone formation are heavily affected by environmental factors, including estrogen, estrogen receptors, and coregulatory proteins, such as the recently reported proline-, glutamic acid-, and leucine-rich protein 1(Pelp1).

**Objective:**

To investigate Pelp1 expression in rat bone mesenchymal stem cells (rBMSCs) during cell proliferation and osteogenic differentiation.

**Methods:**

rBMSCs were cultured in routine and osteogenic differentiation media. Cell proliferation was assessed at days 1, 3, 5, 7, 9, 11, 14, and 21. Pelp1 protein expression in the nucleus and cytoplasm were detected by immunocytochemical analysis. Real-time RT-PCR and western blot were used to detect mRNA and protein expressions of Pelp1, osteocalcin (OCN), and alkaline phosphatase (ALP).

**Results:**

Over 21 days, rBMSCs in routine culture exhibited a 1-2 day lag phase and exponential growth from day 3 to 9, plateauing at day 9, and correlated with temporal mRNA expression of *Pelp1*, which almost reached baseline levels at day 21. In osteogenic induction cultures, *Pelp1* mRNA levels rose at day 9 and steadily increased until day 21, reaching 6.8-fold greater value compared with day 1. Interestingly, *Pelp1* mRNA expression in osteogenic cultures exhibited a trend similar to that of *OCN* expression. *Pelp1* knockdown by siRNA transfection inhibited undifferentiated rBMSC proliferation, and bone markers OCN and ALP expressions in rBMSCs cultured in routine and osteogenic differentiation media.

**Conclusions:**

Pelp1 may be a key player in BMSCs proliferation and osteogenic differentiation, meriting further consideration as a target for development of therapies for pathological bone loss conditions, such as menopausal bone loss.

## Introduction

The metabolic roles of estrogen binding to estrogen receptors (ERs) have been extensively documented in a variety of cells and tissues (such as brain, breast, cardiovascular system, and uterus) [1-3], but their role in osteoblast cell lines has only recently been reported as an important factor in overall bone health [4]. Moreover, current understanding of the osteogenic roles of the numerous cofactors that mediate hormonal effects remains incomplete [4]. In contemporary clinical practice, it is critical to reduce menopausal bone loss, which is often inadequately treated with hormone replacement therapy [5]. Through estrogen, hormonal replacement therapy alter cellular protein and mRNA expression in osteoblastic cells through osteoprotegerin (OPG), receptor activator of NF-κB ligand (RANKL), and ERs, helping to improve matrix mineralization [6,7]. Thus, a better understanding of the roles of estrogen, ERs, and regulatory cofactors in osteogenic processes may form a basis for therapeutic improvements, as well as for the development of novel therapies against menopausal bone loss.

The estrogen-ER complex acts through a series of cell signaling pathways, such as the Src/MAPK cascade, that are highly dependent on regulatory cofactor proteins (coregulators) [8]. While it was previously thought that estrogens act primarily through nuclear ERs, recent reports revealed that rapid estrogen effects involved ERs in the plasma membrane and cytoplasm [8]. Recently, expression of the ER proline-, glutamic acid-, and leucine-rich protein 1 (Pelp1) has been reported in the nucleus and cytoplasm of a wide variety of tissues, most notably the brain [8], mammary gland, ovaries, and uterus [9,10]. It has been suggested that Pelp1 is important for the integration of nuclear receptor (NR) action in both genomic and non-genomic signaling pathways [11]. Thus, Pelp1 may affect signaling pathways that are critical to bone formation and loss.

Environmental cues influenced by coregulatory factors impact the differentiation of undifferentiated multipotent progenitor bone marrow mesenchymal stem cells (BMSCs), which possess notable numbers of ERs α and β, into functional osteoblasts, adipocytes, chondrocytes, myocytes, oligodendrocytes, and neurons [12]. Using *in vivo* murine models, BMSCs implanted on bio-ceramic scaffolds have been successfully used to regenerate bone tissues [13]. The differenciation success may be evaluated using sequential accumulation of collagenous matrix, expression of alkaline phosphatase, secretion of osteocalcin, and bone nodules mineralization [6]. However, the full mechanism of the effects of coregulatory factors, particularly Pelp1, on bone tissue differentiation and growth is not fully documented.

Thus, the aim of this study was to investigate the effects of Pelp1 expression levels on estrogen regulation and on the subsequent proliferation and osteogenic differentiation of BMSCs. A better understanding of the expression profile of Pelp1 in BMSCs during cell growth and osteogenic differentiation may have implications in women’s health after menopause, potentially contributing to the development of new targets for bone tissue restoration therapies.

## Materials and Methods

### 2.1 Cell culture

Routine *in vitro* maintenance cultures were established using sterile frozen finite-lifespan Sprague-Dawley (SD) rat bone marrow mesenchymal stem cells (rBMSCs; RASMX-01001) provided by Cyagen Biosciences, Inc. (Guangzhou, China) [14,15]. The rBMSC nature of these cells was confirmed based on positivity for CD90, CD29, and CD44, and negativity for CD34, CD11b and CD45 [16,17]. Cells were cultured in a humidified incubator with 5% CO_2_ at 37°C in OriCell^TM^ SD Rat Mesenchymal Stem Cell (MSC) Growth Medium without pH indicator (RASMX-90011; Cyagen Biosciences, Inc.), according to the manufacturer’s protocol. SD Rat MSC Growth Medium contained SD Rat MSC basal medium (440 ml), MSC-Qualified fetal bovine serum (FBS) (RASMX -05001-20, Cyagen Biosciences, Inc., 50 ml), penicillin-streptomycin (5 ml), and glutamine (5 ml). Medium was replaced at 3-day intervals.

### 2.2 Osteogenic differentiation

Differentiation into osteoblasts (osteogenesis-induced cells) was achieved by seeding routinely cultured cells at 5×10^3^ cells/cm^2^ in OriCell^TM^ SD Rat MSC Osteogenic Differentiation Medium without pH indicator (RASMX-90021, Cyagen Biosciences, Inc.), according to the manufacturer’s protocol. SD Rat MSC Osteogenic Differentiation Medium contained SD Rat MSC Osteogenic Differentiation Basal Medium, MSC-Qualified FBS (10 ml), penicillin-streptomycin (2 mL), glutamine (2 mL), ascorbate (400 μL), β-glycerophosphate (2 mL), and dexamethasone (20 μL). Medium was replaced at 3-day intervals. 

### 2.3 Pelp1 knockdown by siRNA transient transfection

Pelp1 knockdown was established by blocking *Pelp1* gene expression in rBMSCs cultured in routine and osteogenic differentiation medium using small interfering RNA (siRNA) transfection (Santa Cruz Biotechnology Inc., Hong Kong, China, sc-45287), according to the manufacturer’s protocol. Cells were seeded in 6-well plates and cultured to 60-80% confluence in antibiotic-free BMSC growth medium supplemented with 10% FBS (Cyagen Biosciences, Inc.). Cells were washed once with siRNA Transfection Medium (sc-36868; Santa Cruz Biotechnology Inc.) and the medium was removed. Cells were then transiently transfected with *Pelp1* siRNA or scrambled siRNA (sc-37007; Santa Cruz Biotechnology Inc.) for 72 hours with a mixture of siRNA Transfection Reagent (sc-29528), siRNA Transfection Medium (sc-36868), and siRNA Dilution Buffer (sc-29527) (Santa Cruz Biotechnology Inc.). Transfected cells were used in the subsequent assays. 

### 2.4 Cell proliferation assay

Undifferentiated rBMSCs were plated in triplicate on 24-well culture plates at an initial density of 5×10^3^ cells/well in routine medium. Cells were harvested at days 1, 3, 5, 7, 9, 11, 14, and 21. Typan blue exclusion method combined with CCD imaging were used to calculate the proportion of living cells.

### 2.5 Immunocytochemical assay

Undifferentiated rBMSCs cultured in routine medium were seeded on coverslips at a density of 1×10^4^ cells/cm^2^, cultured for 4 days, and fixed with pre-cooled acetone for 20 min. Immunocytochemical assay was performed using a Streptavidin-Biotin Complex kit (NeoBioscience Technology Co., Ltd. Beijing, China), according to the manufacturer’s instructions. Cells were incubated overnight at 4°C with primary rabbit polyclonal antibody against Pelp1 (ab32912; Abcam, Cambridge, USA) or with the same concentration of normal rabbit serum as a negative control. Pelp1 blocking peptide (ab38389; Abcam, Cambridge, USA) was used to test Pelp1 antibody specificity. Cells were washed three times with phosphate buffered saline (PBS) solution and then incubated with fluorescein isothiocyanate (FITC)-conjugated goat anti-rabbit IgG antibodies (Southern Biotech, Birmingham, USA) for 30 min at room temperature. Cells were then washed again with PBS, and FITC-positive cells were detected by fluorescence microscopy (Olympus CX 41, Tokyo, Japan).

### 2.6 Quantitative real-time RT-PCR

Quantitative real-time RT-PCR (qRT-PCR) was performed to assess Pelp1, osteocalcin (OCN) or alkaline phosphatase (ALP) mRNA expression in osteogenesis-induced and Pelp1 knockdown rBMSCs [18]. Briefly, total RNA was extracted using an EZ 96® Mag-Bind Tissue RNA Kit (Solarbio Technology Co., Shanghai, China), according to the manufacturer’s instructions. Total RNA was quantified, and purity was determined by routine spectrophotometric methods using optical density (OD) values at 260 and 280 nm. First-strand cDNA synthesis was performed using a ReverTrace-α first strand cDNA Synthesis Kit (TOYOBO, Tokyo, Japan). β-actin was used as a normalized control in quantitative analysis. Primers (Genomics Biotech, Beijing, China) were based on the cDNA sequence of rat *Pelp1* (GeneBank Accession No. NM-001024270), OCN (GeneBank Accession No. NM-001032651), ALP (GeneBank Accession No. NM-001201510) and β-actin (Genebank Accession NO. AB-598895). Primer oligonucleotide sequences were: *Pelp1*: forward primer 5’-GGA AGA TGG CGG CAG CCG TT-3’, reverse primer 5’-TCA CTG CCG AGA GAC CCC CG-3’; OCN: forward primer 5’-CAG ACA AGT CCC ACA CAG CA-3’, reverse primer 5’-CTT TAT TTT GGA GCT GCT GT-3’; ALP: forward primer 5’-GCC CTC TCC AAG ACA TAT A-3’, reverse primer 5’-CCA TGA TCA CGT CGA TAT CC-3’; β-actin: forward primer 5’-ACC TTC TAC AAT GAG CTG CG-3’, reverse primer 5’-CCT GGA TAG CAA CGT ACA TGG-3’. qRT-PCR was performed using cDNA and SYBR green PCR master mix (Genolab Biotech, Beijing, China) together with primer pairs in an ABI Prism 7100 system (Applied Biosystems, Carlsbad, USA). Transcript quantification was performed in triplicate and reported relatively to β-actin. Pre-denaturation was conducted at 95°C for 5 min, and then using 40 cycles of 95°C for 30 s, 65°C for 30 s, and 72°C for 2 min. The Ct value was defined as the number of PCR cycles in which the fluorescence signal exceeded the detection threshold value. Data for each sample was then analyzed using the 2^-ΔΔCt^ method [6]: ΔCt_Target protein (day n)_ = Ct_Target protein (day n)_ - Ct _β-actin (day n)_, ΔΔCt = ΔCt_Target protein (day n)_ - ΔCt _Target protein (day 1)_. Lastly, 2^-ΔΔCt^ was calculated to represent the relative mRNA expression of target genes. 

### 2.7 Western blotting

Cells were washed twice with PBS and lysed on ice for 10 min in radio-immunoprecipitation assay (RIPA) buffer with 10:1 phenylmethylsulfonyl fluoride (PMSF) (NeoBioscience Technology Co., Ltd. Beijing, China). Lysates were centrifuged in an Eppendorf centrifuge (12000 r/min) at 4°C for 15 min, and protein was quantified by the Bradford method. Cells lysates containing equal amounts of protein (50 μg) were collected, separated on 6% sodium dodecyl sulfate polyacrylamide gel electrophoresis (SDS-PAGE) gels, and transferred onto nitrocellulose (NC) membranes (NeoBioscience Technology Co., Ltd. Beijing, China). Membranes were blocked for 1 h in a blocking buffer (Sigma-Aldrich Co., Shanghai, China) containing 5% powdered milk, and were immunoblotted overnight at 4°C with primary rabbit anti-mouse antibody against Pelp1 (1:1000; Abcam Biotechnology, Inc., Cambridge, USA), ALP (1:1000; Beyotime, Beijing, China), OCN (1:1000; Beyotime, Beijing, China) and β-actin (1:1000; Beyotime, Beijing, China). Membranes were then incubated with horseradish peroxidase (HRP)-conjugated goat anti-rabbit secondary antibody (Southern Biotech, Birmingham, USA). Specific bands were detected by enhanced chemiluminescence, and bands were quantified using Sigma Gel-Gel Analysis software (Sigma-Aldrich, St Louis, MI, USA), as previously described [19].

### 2.8 Statistical analysis

All experiments were performed in triplicate. Data were expressed as means ± standard deviation (SD) for each group. All data were analyzed using SPSS 12.0 (SPSS Inc., Chicago, USA). Statistical significance was evaluated using the Student’s *t*-test or one-way analysis of variance (ANOVA) with the Dunn multiple comparison test. *P*-values <0.05 were considered statistically significant (*P* < 0.05).

## Results

### 3.1 Pelp1 protein expression in undifferentiated rBMSCs

**Figure 1:**
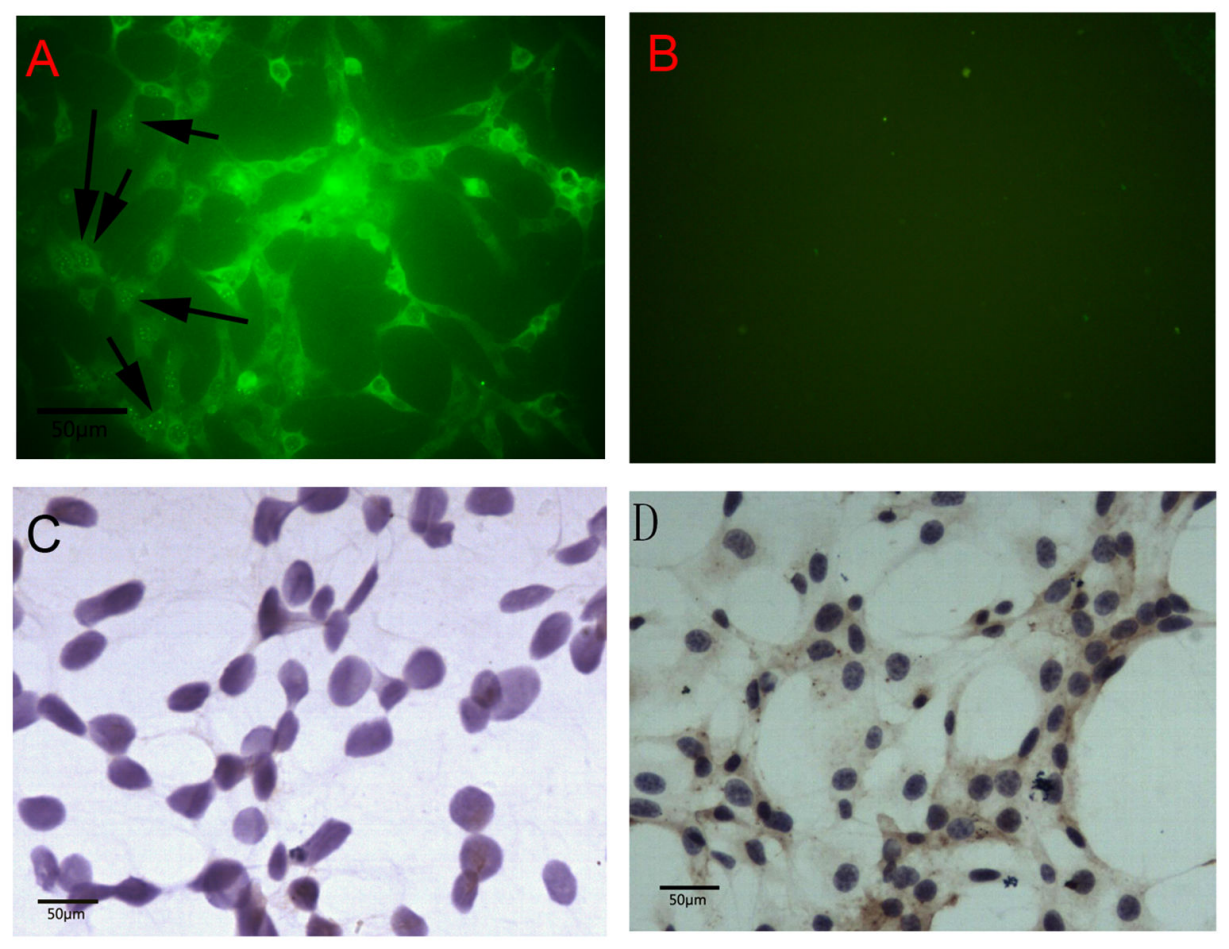
Expression of Pelp1 protein in rat bone marrow mesenchymal stem cells (rBMSCs) cultured *in* *vitro* for 4 days. (A) Immunofluorescence staining (×200) showed that Pelp1 was predominantly localized in cytoplasm rather than nuclei in undifferentiated rBMSCs. The arrows show Pelp1-positive staining localized in nuclei. (B) Isotype control. (C) Immunocytochemistry staining (×200) showed Pelp1-negative staining in rBMSCs using Pelp1 blocking peptide. (D) Immunocytochemistry staining (×200) showed Pelp1 positive staining in rBMSCs using Pelp1 antibody.

Immunofluorescence staining revealed that Pelp1 protein was localized in both the nuclei and cytoplasm of undifferentiated rBMSCs, but was predominantly localized in the cytoplasm (Figure 1A). Pelp1 positive staining in undifferentiated rBMSCs using Pelp1 antibody produced consistent results (Figure 1D). When subjected to Pelp1 antigen blocking as control, no Pelp1 expression was apparent in undifferentiated rBMSCs (Figure 1C). 

### 3.2 Temporal mRNA and protein expressions of Pelp1 in undifferentiated rBMSCs

**Figure 2:**
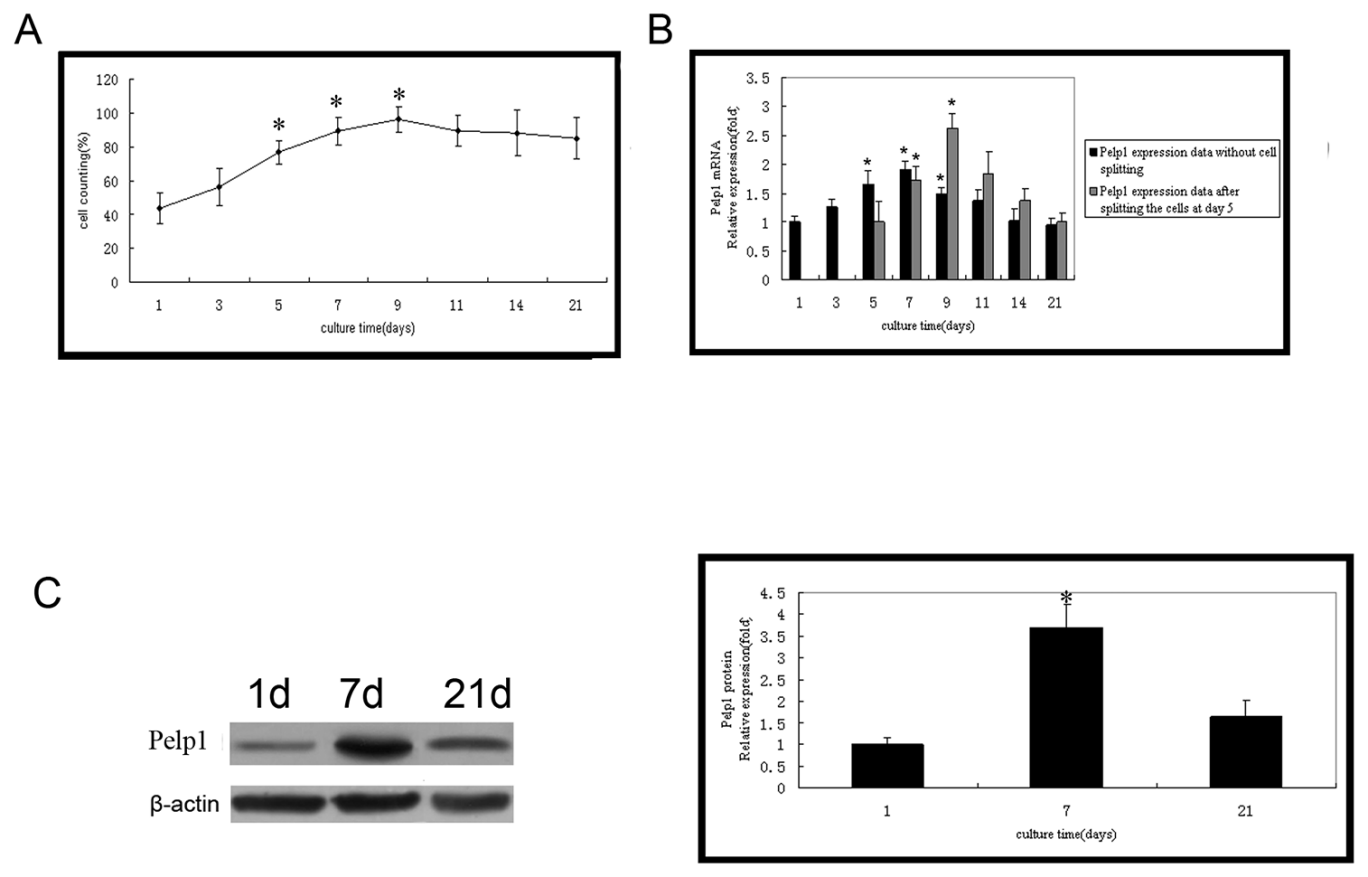
mRNA and protein expressions of Pelp1 in time in undifferentiated rBMSCs during a 21-day period. (A) Growth curve of undifferentiated rBMSCs. (B) Temporal changes in Pelp1mRNA expression were determined qRT-PCR. β-actin was used as an internal control. Pelp1 mRNA expression without splitting the cells was assessed at days 1, 3, 5, 7, 9, 11, 14 and 21 (left panel). *P<0.05 *vs*. baseline value (day 1). In another experiment, rBMSCs were split at day 5 to prevent them from reaching confluence at day 7, and Pelp1 mRNA expression was assessed at days 7, 9, 11 and 14 (right panel). ^#^ *P*<0.05 *vs*. value at day 5. (C) Temporal changes in Pelp1 protein expression were determined by western blotting. β-actin was used as an internal control. Data represent the mean ± SD of three independent experiments. *P<0.05 *vs*. baseline value (day 1).

Over 21 days, rBMSCs in routine culture exhibited a 1-2 day lag phase and exponential growth from days 3 to 9, plateauing at day 9 (Figure 2A). The temporal mRNA expression of *Pelp1* assessed by qRT-PCR during the exponential growth period (day 3-7) increased steadily, peaking at day 7 (Figure 2B). Notably, this peak in Pelp1 expression correlated with the cells reaching confluence. *Pelp1* expression decreased after plateauing at day 9 (Figure 2B, left panel). To confirm this, rBMSCs were split at day 5 to prevent them from reaching confluence at day 7, and Pelp1 expression was assessed at days 7, 9, 11 and 14. Results showed that Pelp1 expression continued to increase after day 7, peaking at day 9 when confluence was reached (Figure 2B, right panel). Western blotting confirmed these results (Figure 2C). Pelp1 expression almost returned to baseline (day 1) levels by day 21. 

### 3.3 Pelp1 and bone markers expressions in osteogenesis-induced rBMSCs

**Figure 3:**
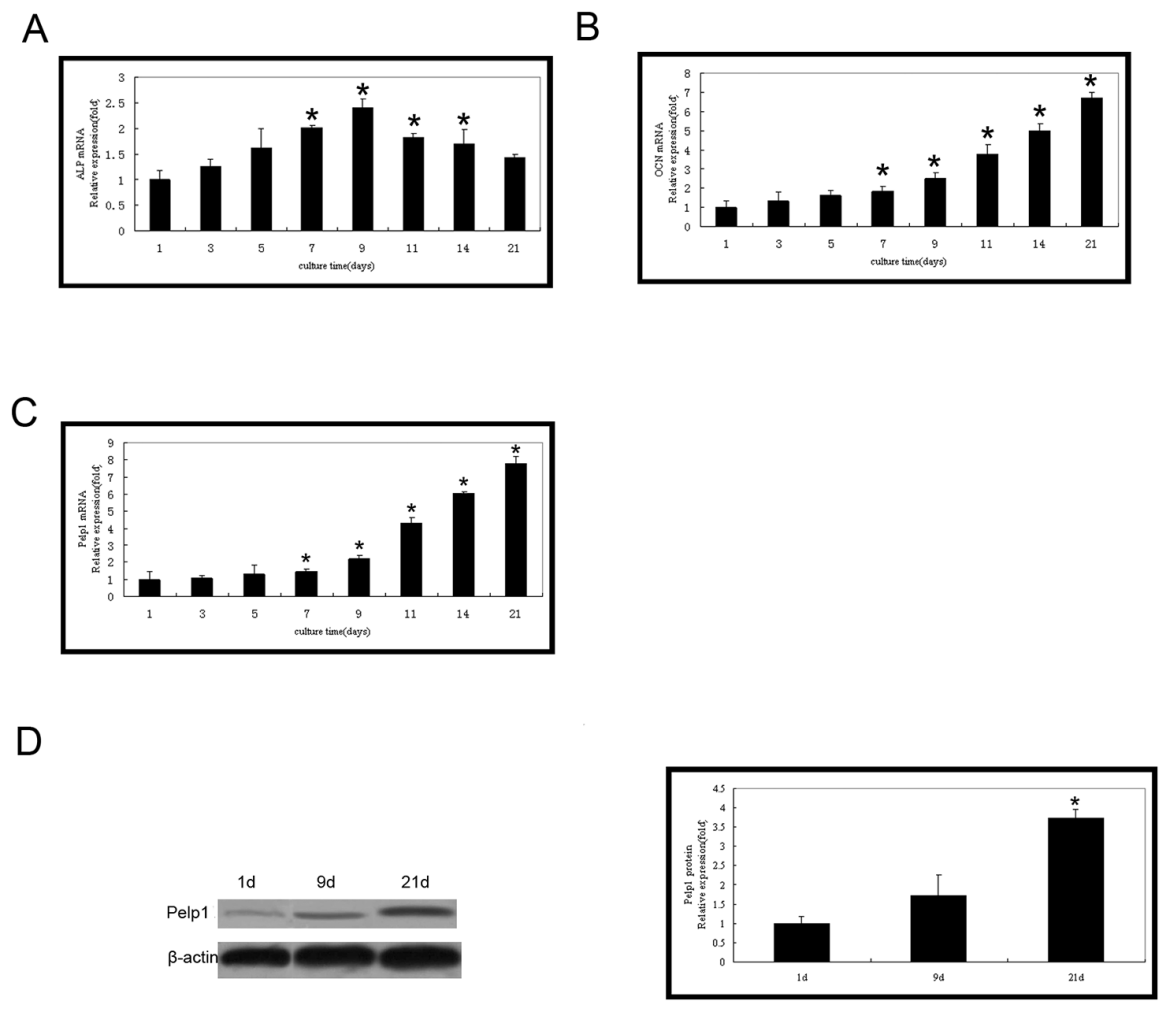
Temporal expression of Pelp1 and bone markers in rBMSCs during osteogenic differentiation. rBMSCs were cultured in osteogenic differentiation culture for 1, 3, 5, 7. 9. 11. 14, and 21 days. mRNA expressions of ALP (A), OCN (B) and Pelp1 (C) were examined by real-time RT-PCR. β-actin was used as an internal control. (D) Protein expression of Pelp1 during osteogenic differentiation was examined by western blotting. β-actin was used as an internal control. The mRNA and protein expressions at 1 day of culture were normalized to “1”. Data represent the mean ± SD of three independent experiments. *P<0.05 *vs*. baseline value (day 1).

The bone markers *ALP* and *OCN* were both highly expressed in osteogenesis-induced rBMSCs. *ALP* mRNA expression increased from days 1 to 9 and peaked at day 9 (Figure 3A). In contrast, *OCN* mRNA expression was up-regulated after day 7 (Figure 3B). The *Pelp1* mRNA expression in osteogenesis-induced rBMSCs was significantly different compared with the routine culture (Figure 3C) *Pelp1* mRNA levels were low during the early stage (from day 1 to 7) of osteogenic induction compared with those of the routine culture, and *Pelp1* mRNA levels increased by day 9 and steadily increased until day 21. By day 21, *Pelp1* mRNA expression was significantly 6.8-fold greater than baseline (day 1) value (*P* < 0.05). Interestingly, the pattern of *Pelp1* mRNA expression in osteogenic cultures was similar to *OCN* expression pattern (Figure 3B). Furthermore, western blotting indicated consistent findings in Pelp1 protein expression (Figure 3D).

### 3.4 *Pelp1* knockdown inhibited mRNA and protein expressions of Pelp1 and affected undifferentiated rBMSC growth

**Figure 4:**
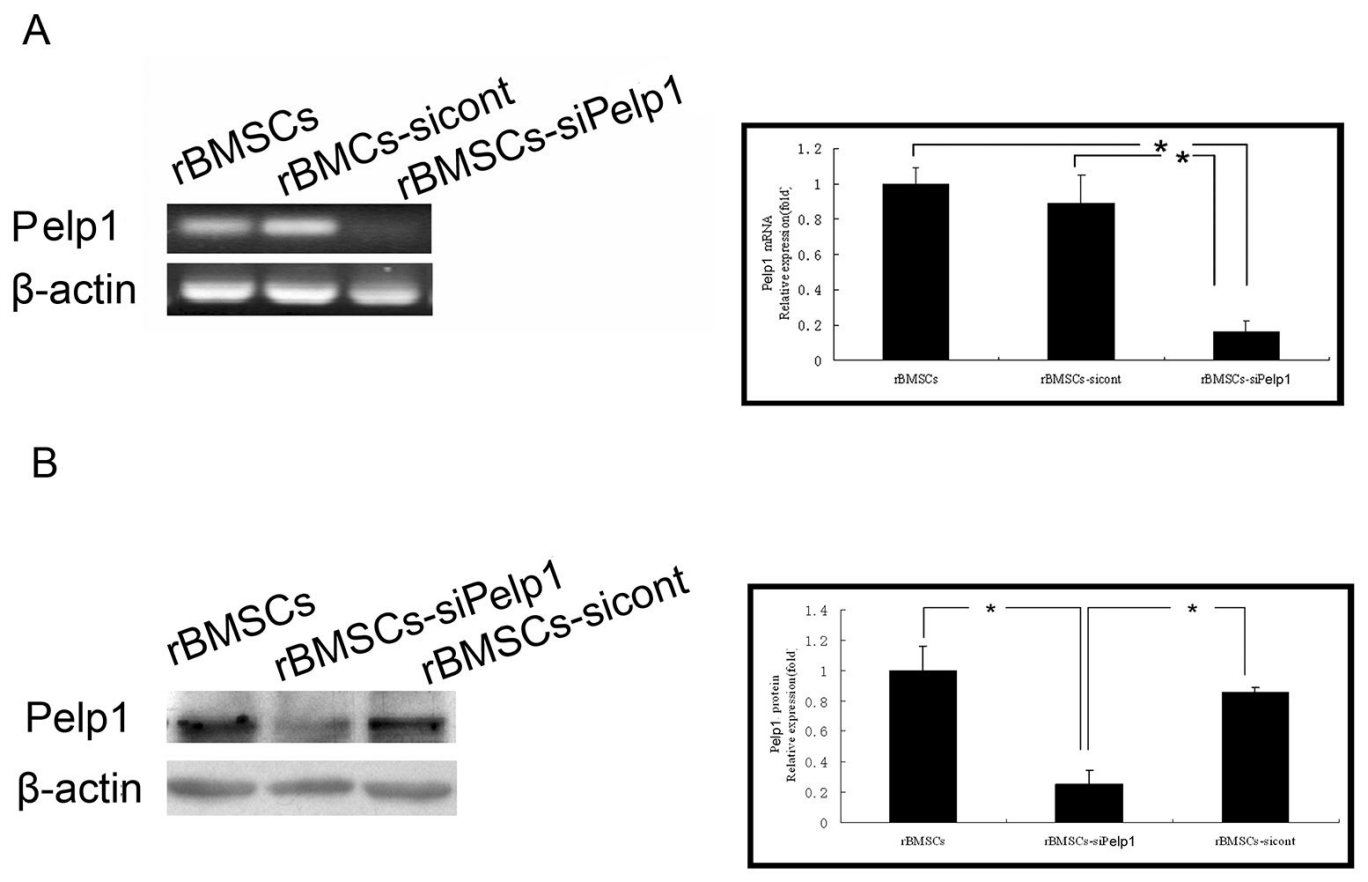
Suppression of Pelp1 mRNA and protein expressions in undifferentiated rBMSCs by si-Pelp1 transfection after 72h. (**A**) **Pelp1 mRNA expression was determined by qRT-PCR**. β-actin was used as an internal control. (B) Pelp1 protein expression was determined by western blotting. β-actin was used as an internal control. Data represent the mean ± SD of three independent experiments. *P<0.05, *vs*. rBMSCs-siPelp1.

Pelp1 mRNA and protein expressions were detected in undifferentiated rBMSCs transfected with si-Pelp1 (rBMSCs-siPelp1) or scramble siRNA (rBMSCs-sicont) by qRT-PCR (Figure 4A) and western blotting (Figure 4B). Compared with the rBMSCs-sicont group, si-Pelp1 transfection significantly inhibited Pelp1 mRNA and protein expressions by 84% and 75%, respectively (both *P*<0.05). However, there were no significant differences in Pelp1 mRNA and protein expressions between the rBMSCs-sicont and the rBMSCs groups (both *P*>0.05). 

**Figure 5:**
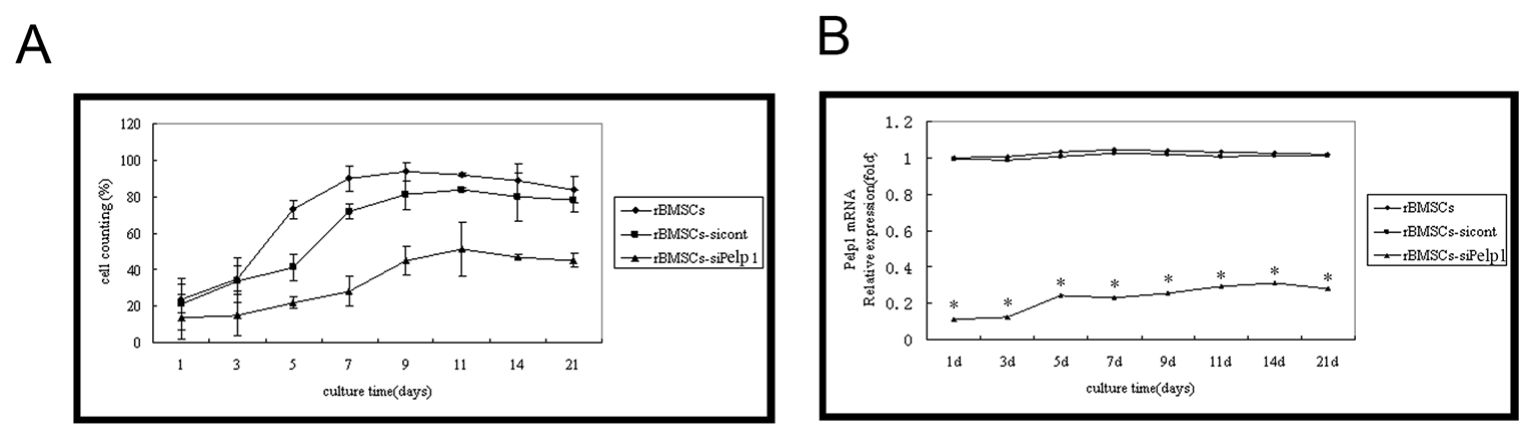
*Pelp1* knockdown affects undifferentiated rBMSC growth. (A) Growth curves of undifferentiated rBMSCs after transfection with si-Pelp1 or scramble siRNA for 1, 3, 5, 7, 9, 11, 14, and 21 days. (B) Temporal changes in relative *Pelp1* mRNA expression after transfection with si-Pelp1 or scramble siRNA for 1, 3, 5, 7, 9, 11, 14, and 21 days, by qRT-PCR. β-actin was used as an internal control. Data represent the mean ± SD of three independent experiments. *P<0.05 rBMSCs-siPelp1 vs. rBMSCs-sicont.

Compared with the rBMSCs-sicont group, si-Pelp1 transfection significantly inhibited undifferentiated rBMSC growth (all *P*<0.05) (Figure 5A). However, there were no significant differences in undifferentiated rBMSC growth between the rBMSCs-siPelp1 and the rBMSCs groups (all *P*>0.05). Results of *Pelp1* mRNA expression correlated with cell confluence (Figure 5B). 

### 3.5 Effect of si-Pelp1 transfection on the expressions of bone markers in rBMSCs during osteogenic differentiation

**Figure 6:**
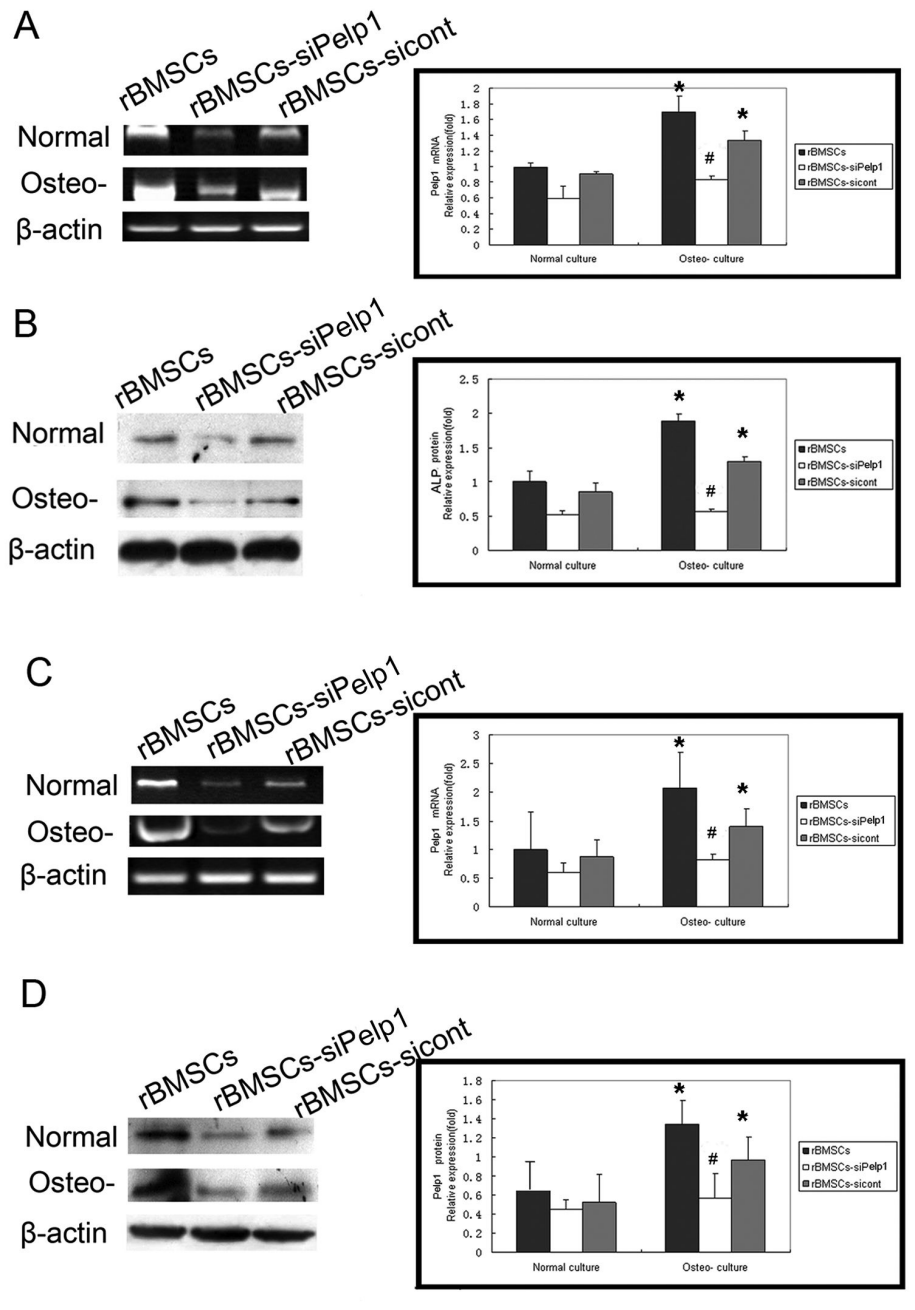
Effects of si-Pelp1 transfection on the expression of bone markers in rBMSCs during osteogenic differentiation. si-Pelp1 and scramble siRNA were transfected into rBMSCs cultured in normal and osteogenic differentiation culture. After 3 days of transfection, mRNA expression of ALP (A) and OCN (C) was examined by qRT-PCR and protein expression of ALP (B) and OCN (D) was examined by western blotting. ALP and OCN expression was quantified by Gray-scale analysis. Data represent the mean ± SD of three independent experiments. *P<0.05, normal culture *vs*. osteogenic differentiated culture; ^#^ *P*<0.05 rBMSCs-siPelp1 *vs*. rBMSCs-sicont. Normal: normal culture; osteo-: osteogenic differentiation culture .

*Pelp1* knockdown significantly decreased ALP (Figure 6A and 6B) and OCN (Figure 6C and 6D) mRNA and protein expressions in rBMSCs cultured in routine and osteogenic differentiation media, compared with the rBMSCs-sicont group (all *P*<0.05). There were no significant differences in ALP and OCN mRNA and protein expressions between undifferentiated and differentiated rBMSCs in the rBMSCs-siPelp1 group (all *P*>0.05). However, in the rBMSCs and rBMSCs-sicont groups, ALP and OCN mRNA and protein expressions in the differentiated rBMSCs were significantly higher than in the undifferentiated rBMSCs (all *P*<0.05). 

## Discussion

Pelp1 is a regulator of estrogen and its receptor, and may play a role in bone formation. Indeed, results from the present study suggest that undifferentiated rBMSCs, which are cells targeted by estrogen and involved in bone development, express Pelp1 in their cytoplasm and nuclei, but mainly in their cytoplasm, suggesting a decreased osteogenesis since Pelp1 has to localize to the nucleus to exert its genomic effects [9,10,20]. Furthermore, rBMSCs cultured in routine medium exhibited increased Pelp1 expression during the linear proliferative stage, but Pelp1 expression gradually decreased after cells reached confluence. These *in vitro* findings suggest that Pelp1 may participate in the regulation of osteogenesis *in vivo*, though further study will be required to explore potential clinical implications of Pelp1 protein and *Pelp1* gene regulation as a therapeutic target, as in the case of menopausal bone loss.

The importance of Pelp1 in osteogenesis demonstrated by the current study is consistent with previous indications that ER-α is expressed in BMSCs, where it plays roles in regulating DNA synthesis, cell proliferation, and cell death [6]. Though many different factors are involved in bone formation [21], estrogen and its receptors are generally regarded as the predominant environmental factors for osteogenesis induction and formation [22], although the complete mechanisms involved still require study. Thus, it is likely that Pelp1 levels may indirectly affect non-genomic estrogen pathways mediated by ER-α, interacting with numerous growth factors pathways, and potentially causing pathological alterations in bone formation [23]. These non-genomic events may have important physiological consequences, and they may be useful in engineering new therapies to alleviate pathological bone loss.

The function of intracellular Pelp1 has been shown to be a result of both its expression levels and its cellular localization [11], which has important implications for Pelp1 in BMSCs, which predominantly exhibited Pelp1 in their cytoplasm. In the brain, Pelp1 has been reported to co-localize in the nucleus with ER-α, though some cytoplasmic and plasma membrane-associated Pelp1 is also common in most reports [20]. In salivary duct carcinoma, Pelp1 was reported to be localized in the cytoplasm. In epithelial, endothelial, and smooth muscle cells, Pelp1 was observed in cell nuclei along with ER-β [24]. In the present study, because Pelp1 was predominantly localized in the cytoplasm, Pelp1 expression is more likely to be cell-specific and, thereby, play a crucial role in estrogen signaling pathways on an individual cell basis rather than on the whole tissue [11,19,25,26]. Due to the documented role of Pelp1 in non-genomic estrogen and ER pathways involved in cell survival, apoptosis and, estrogen-induced osteo-, neuro-, and cardio-protection [27], Pelp1 may also participate in regulating these vital cell functions in BMSCs in individual cells, thereby contributing to different bone regulation and growth in different tissues of the body.

In osteogenesis, BMSCs are important precursor cells that rely on estrogen for cell growth and proliferation [28,29]. Thus, BMSCs undergoing osteogenic commitment may be divided based on bone markers, as previously described [6]. Using this system, it is simple to distinguish the proliferation and commitment stages by increasing cell proliferation, the differentiation phase by ALP up-regulation, and the maturation stage by induction of mineralization and OCN expression [6]. The findings of the current study further suggest that Pelp1 expression may be useful in classifying these cells due to its increase at day 7 in osteogenic-induced BMSCs, coincident with increases in OCN. Thus, further research will be required to explore potential prognostic and diagnostic utilities of Pelp1 levels, based on the potential of Pelp1 to increase BMSC osteogenic differentiation.

However, the current study is limited by its *in vitro* nature, requiring further confirmation using *in vivo* models. While evaluation of the clinical potential of Pelp1 regulation requires much more extensive research, the current study provides sound preliminary indications that Pelp1 protein levels and *Pelp1* gene activation may play a role in osteogenesis and, potentially, in pathological bone conditions, such as menopausal bone loss. Notably, inhibited cell growth and osteogenesis in *Pelp1* knockdown cells were observed in the current study, supporting this hypothesis. However, it must be considered that the first phase of osteogenic differentiation is cell proliferation; thus, osteogenic induction cannot occur because initial cell division is blocked, rather than because Pelp1 is actually required for osteogenic differentiation. Thus, further study will be required to confirm the effects of Pelp1 on osteogenic differentiation.

Pelp1 expression in rBMSCs was up-regulated during cell proliferation and late stage of differentiation in osteogenesis-induced rBMSCs. Furthermore, Pelp1 knockdown inhibited cell proliferation and osteogenic differentiation in rBMSCs. These results demonstrate that Pelp1, as an estrogen co-regulator, may participate in rBMSC cell proliferation and differentiation, meriting further consideration as a target for development of therapies for pathological bone loss conditions, such as menopausal bone loss.
