# Correction: Temporal Expression of Pelp1 during Proliferation and Osteogenic Differentiation of Rat Bone Marrow Mesenchymal Stem Cells

**DOI:** 10.1371/annotation/5fa9cfb4-9964-4586-845d-d8205f318d68

**Published:** 2013-12-16

**Authors:** Jing Wang, Shujun Song, Liang Shi, Qiang Zhu, Chuanchuan Ma, Xiaoqing Tan, Yin Ding, Zhongying Niu

The first and second authors, Jing Wang and Shujun Song, respectively, should be indicated as having contributed equally to the paper. In addition, affiliation number 1 for the first and seventh authors. The correct affiliation is: Department of Orthodontics, College of Stomatology, Fourth Military Medical University, Xin Cheng District, Xi’an, China. 

