# Correction: Temporal Expression of Pelp1 during Proliferation and Osteogenic Differentiation of Rat Bone Marrow Mesenchymal Stem Cells

**DOI:** 10.1371/annotation/b5254278-c64f-40e1-90a8-de2ac7ffc001

**Published:** 2013-12-30

**Authors:** Jing Wang, Shujun Song, Liang Shi, Qiang Zhu, Chuanchuan Ma, Xiaoqing Tan, Yin Ding, Zhongying Niu

The Authors are listed out of order, and Zhu Qiang, the author currently appearing as the fourth author, should be indicated as contributing equally to the article with the first author, Jing Wang. Please view the correct author order, affiliations, and citation here: Jing Wang^1^, Zhu Qiang^2^, Shujun Song ^3^, Liang Shi ^4^, Chuanchuan Ma^4^, Xiaoqing Tan^3^, Din Ying^1^, Zhongying Niu^4^.

1 Department of Orthodontics, College of Stomatology, Forth Military Medical University, Xin Cheng District, Xi’an, China

2 Department of Urological, General Hospital of People’s Liberation Army, Hai Dian District, Beijing, China

3 Department of Pathology and Experimental Medicine, 306 Hospital of PLA, Chao Yang District, Beijing, China

4 Department of Stomatology, 306 Hospital of PLA, Chao Yang District, Beijing, China.

Wang J, Zhu Q, Song S, Shi L, Ma C, et al. (2013) Temporal Expression of Pelp1 during Proliferation and Osteogenic Differentiation of Rat Bone Marrow Mesenchymal Stem Cells. PLoS ONE 8(10): e75477. doi:10.1371/journal.pone.0075477.

