# Correction: Temporal Expression of Pelp1 during Proliferation and Osteogenic Differentiation of Rat Bone Marrow Mesenchymal Stem Cells

**DOI:** 10.1371/journal.pone.0090259

**Published:** 2014-02-18

**Authors:** The PLOS ONE Staff

There was an error in the Formal Correction posted on December 30, 2013. The name of the seventh author was misspelled in the correction. The correct name is Ding Yin.
